# 4-Amino­pyridinium 2-chloro-4-nitro­benzoate monohydrate

**DOI:** 10.1107/S1600536812046077

**Published:** 2012-11-17

**Authors:** Kelsey L. Savig, Andreas Lemmerer

**Affiliations:** a Molecular Sciences Institute, School of Chemistry, University of the Witwatersrand, Johannesburg, PO Wits 2050, South Africa

## Abstract

In the title hydrated mol­ecular salt, C_5_H_7_N_2_^+^·C_7_H_3_ClNO_4_^−^·H_2_O, the ions and water mol­ecules assemble into ribbons of *R*_6_^5^(22) rings along the *c* axis *via* O(water)—H⋯O^−^, N^+^—H⋯O(water) and N—H⋯O^−^ hydrogen bonds. N—H⋯O^−^ hydrogen bonds connect adjacent ribbons along the *c*-axis direction *via R*_4_^4^(12) rings, forming hydrogen-bonded layers. The CO_2_ and NO_2_ groups make dihedral angles of 81.8 (2) and 1.4 (2)°, respectively, with the ring in the anion.

## Related literature

For related structures, see: Lemmerer *et al.* (2010 ▶). For graph-set notation, see: Bernstein *et al.* (1995 ▶). 
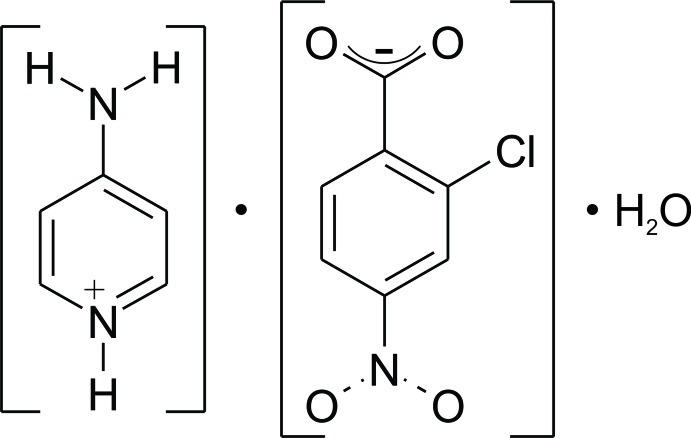


## Experimental

### 

#### Crystal data

C_5_H_7_N_2_^+^·C_7_H_3_ClNO_4_^−^·H_2_O
*M* *_r_* = 313.7
Monoclinic, 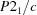

*a* = 14.4500 (5) Å
*b* = 14.3300 (5) Å
*c* = 6.9918 (2) Å
β = 97.804 (2)°
*V* = 1434.37 (8) Å^3^
*Z* = 4
Mo *K*α radiation
μ = 0.29 mm^−1^
*T* = 173 K
0.78 × 0.31 × 0.09 mm

#### Data collection

Bruker APEXII CCD area-detector diffractometer
Absorption correction: integration (*XPREP*; Bruker, 2004 ▶) *T*_min_ = 0.896, *T*_max_ = 0.977
14928 measured reflections
3456 independent reflections
2685 reflections with *I* > 2σ(*I*)
*R*_int_ = 0.061

#### Refinement

*R*[*F* ^2^ > 2σ(*F* ^2^)] = 0.043
*wR*(*F* ^2^) = 0.122
*S* = 1.04
3456 reflections
210 parameters
H atoms treated by a mixture of independent and constrained refinement
Δρ_max_ = 0.5 e Å^−3^
Δρ_min_ = −0.36 e Å^−3^

### 

Data collection: *APEX2* (Bruker, 2005 ▶); cell refinement: *SAINT-Plus* (Bruker, 2004 ▶); data reduction: *SAINT-Plus* and *XPREP* (Bruker 2004 ▶); program(s) used to solve structure: *SHELXS97* (Sheldrick, 2008 ▶); program(s) used to refine structure: *SHELXL97* (Sheldrick, 2008 ▶); molecular graphics: *ORTEP-3 for Windows* (Farrugia, 2012 ▶) and *DIAMOND* (Brandenburg, 1999 ▶); software used to prepare material for publication: *WinGX* (Farrugia, 2012 ▶) and *PLATON* (Spek, 2009 ▶).

## Supplementary Material

Crystal structure: contains datablock(s) global, I. DOI: 10.1107/S1600536812046077/fy2073sup1.cif

Structure factors: contains datablock(s) I. DOI: 10.1107/S1600536812046077/fy2073Isup2.hkl

Supplementary material file. DOI: 10.1107/S1600536812046077/fy2073Isup3.mol

Supplementary material file. DOI: 10.1107/S1600536812046077/fy2073Isup4.cml

Additional supplementary materials:  crystallographic information; 3D view; checkCIF report

## Figures and Tables

**Table 1:** Hydrogen-bond geometry (Å, °)

*D*—H⋯*A*	*D*—H	H⋯*A*	*D*⋯*A*	*D*—H⋯*A*
N2—H2⋯O1*W* ^i^	0.93 (3)	1.72 (3)	2.641 (2)	170 (2)
N3—H3*A*⋯O1	0.94 (2)	1.99 (2)	2.926 (2)	171.1 (19)
N3—H3*B*⋯O2^ii^	0.84 (2)	2.16 (3)	2.982 (2)	165 (2)
O1*W*—H1*W*⋯O1^iii^	0.75 (3)	1.96 (3)	2.698 (2)	166 (3)
O1*W*—H2*W*⋯O2	0.78 (4)	1.99 (4)	2.729 (2)	158 (3)

Symmetry codes: (i) 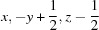; (ii) 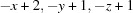; (iii) 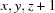.

**Fig. 1.**
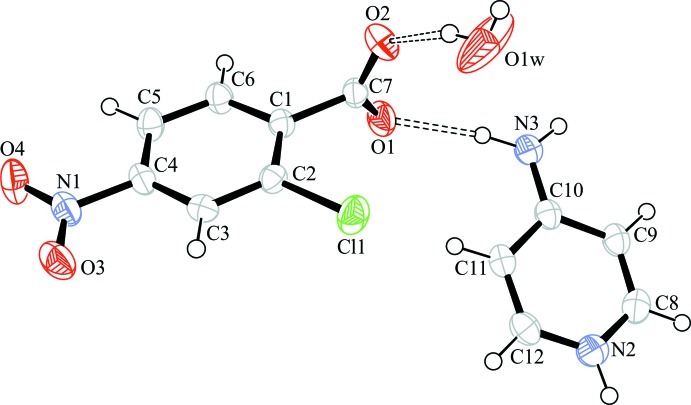
The asymmetric unit of (I) showing the atomic numbering scheme. Displacement ellipsoids are shown at the 50% probability level.

**Fig. 2.**
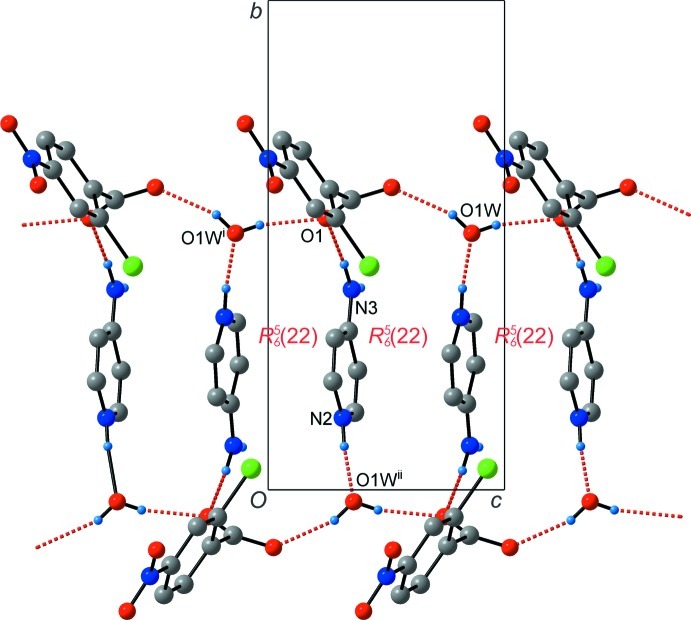
Hydrogen bonding diagram of (I) showing the R56(22) rings to form ribbons. Intermolecular hydrogen bonds are shown as dashed red lines forming dimers. Atoms with superscrips (i) and (ii) are at the symmetry positions (x, y, z - 1) and (x, -y + 1/2, z - 1/2) respectively.

**Fig. 3.**
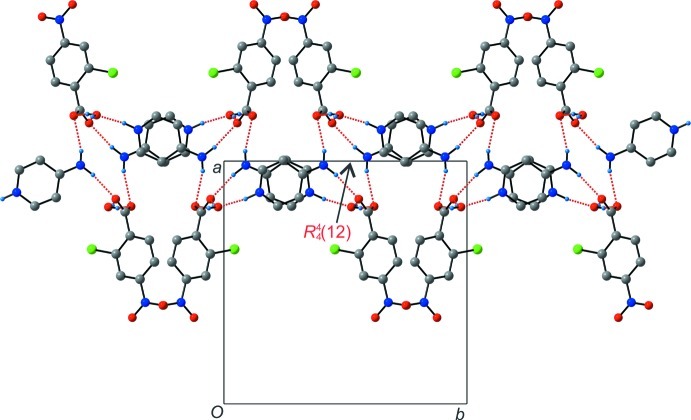
The layers form by combining the one-dimensional ribbons using R44(12) rings.

## supplementary crystallographic information

### Comment

The title molecular salt is part of a larger research project looking at the factors that determine if a salt or a co-crystal forms between a specific carboxylic acid and various pyridine ring systems (Lemmerer *et al.*, 2010).

In molecular salt (I) (Fig. 1), formed by dissolving 4-aminopyridine and 2-chloro-4-nitrobenzoic acid in methanol, two kinds of hydrogen-bonded rings are formed. The ring *R*^5^_6_(22) (Bernstein *et al.*, 1995) uses O1W—H···O^-^, N^+^—H···O and N—H···O^-^ hydrogen bonds to connect all three species together into a 1-D ribbon (Fig. 2) along the *c*-axis. In addition, a *R*^4^_4_(12) ring is formed between two amine groups and two carboxylate groups to connect two ribbons together to form layers (Fig. 3).

### Experimental

Crystals were grown by slow evaporation of a methanol solution of 2-chloro-4-nitrobenzoic acid (0.100 g; 0.496 mmol) and 4-aminopyridine (0.047 g; 0.50 mmol) in 8 ml of methanol, and afforded colourless plates after three days of slow evaporation at ambient conditions.

### Refinement

The aromatic C-bound H atoms were geometrically placed with C—H bond lengths of 0.95 Å, and were refined as riding with *U*_iso_(H) = 1.2*U*_eq_(C). The O-bound H atoms of the water molecule and the N-bound H atoms were located in the difference map and their coordinates as well as isotropic displacement parameters were refined freely. The residual electron density around the O atom of the water molecule was found to not correspond to any H atoms by inspection of the hydrogen bonding geometry.

### Crystal data

**Table:**

C_5_H_7_N_2_^+^·C_7_H_3_ClNO_4_^−^·H_2_O	*F*(000) = 648
*M**_r_* = 313.7	*D*_x_ = 1.453 Mg m^−^^3^
Monoclinic, *P*2_1_/*c*	Mo *K*α radiation, λ = 0.71073 Å
Hall symbol: -P 2ybc	Cell parameters from 4262 reflections
*a* = 14.4500 (5) Å	θ = 2.8–27.8°
*b* = 14.3300 (5) Å	µ = 0.29 mm^−^^1^
*c* = 6.9918 (2) Å	*T* = 173 K
β = 97.804 (2)°	Plate, colourless
*V* = 1434.37 (8) Å^3^	0.78 × 0.31 × 0.09 mm
*Z* = 4	

### Data collection

**Table:**

Bruker APEXII CCD area-detector diffractometer	2685 reflections with *I* > 2σ(*I*)
ω scans	*R*_int_ = 0.061
Absorption correction: integration (*XPREP*; Bruker, 2004)	θ_max_ = 28°, θ_min_ = 1.4°
*T*_min_ = 0.896, *T*_max_ = 0.977	*h* = −19→19
14928 measured reflections	*k* = −18→18
3456 independent reflections	*l* = −8→9

### Refinement

**Table:**

Refinement on *F*^2^	0 restraints
Least-squares matrix: full	H atoms treated by a mixture of independent and constrained refinement
*R*[*F*^2^ > 2σ(*F*^2^)] = 0.043	*w* = 1/[σ^2^(*F*_o_^2^) + (0.0662*P*)^2^ + 0.1397*P*] where *P* = (*F*_o_^2^ + 2*F*_c_^2^)/3
*wR*(*F*^2^) = 0.122	(Δ/σ)_max_ < 0.001
*S* = 1.04	Δρ_max_ = 0.5 e Å^−^^3^
3456 reflections	Δρ_min_ = −0.36 e Å^−^^3^
210 parameters	

### Special details

**Table:**

Experimental. Numerical integration absorption corrections based on indexed crystal faces were applied using the *XPREP* routine (Bruker, 2004)
Geometry. All e.s.d.'s (except the e.s.d. in the dihedral angle between two l.s. planes) are estimated using the full covariance matrix. The cell e.s.d.'s are taken into account individually in the estimation of e.s.d.'s in distances, angles and torsion angles; correlations between e.s.d.'s in cell parameters are only used when they are defined by crystal symmetry. An approximate (isotropic) treatment of cell e.s.d.'s is used for estimating e.s.d.'s involving l.s. planes.

### Fractional atomic coordinates and isotropic or equivalent isotropic displacement parameters (Å^2^)

**Table:**

	*x*	*y*	*z*	*U*_iso_*/*U*_eq_	
C1	0.69246 (11)	0.61389 (11)	0.2594 (2)	0.0261 (3)	
C2	0.61757 (12)	0.55610 (11)	0.2858 (2)	0.0271 (3)	
C3	0.52758 (11)	0.57500 (11)	0.2008 (2)	0.0284 (3)	
H3	0.4772	0.5348	0.2188	0.034*	
C4	0.51347 (11)	0.65438 (12)	0.0887 (2)	0.0290 (4)	
C5	0.58469 (12)	0.71419 (12)	0.0592 (2)	0.0325 (4)	
H5	0.5725	0.7687	−0.0176	0.039*	
C6	0.67437 (12)	0.69317 (12)	0.1438 (2)	0.0311 (4)	
H6	0.7245	0.7332	0.1232	0.037*	
C7	0.79130 (11)	0.59307 (11)	0.3515 (2)	0.0291 (3)	
N1	0.41775 (10)	0.67644 (11)	0.0004 (2)	0.0349 (3)	
O1	0.84325 (9)	0.55490 (9)	0.24589 (19)	0.0412 (3)	
O2	0.81313 (9)	0.61801 (10)	0.52270 (17)	0.0424 (3)	
O3	0.35519 (9)	0.62315 (10)	0.0282 (2)	0.0462 (3)	
O4	0.40575 (10)	0.74821 (11)	−0.0962 (2)	0.0513 (4)	
Cl1	0.63660 (3)	0.45679 (3)	0.42817 (7)	0.04119 (15)	
C8	0.96351 (14)	0.15836 (13)	0.3659 (2)	0.0361 (4)	
H8	1.0019	0.1049	0.3936	0.043*	
C9	1.00312 (12)	0.24412 (13)	0.3780 (2)	0.0341 (4)	
H9	1.0687	0.2506	0.4123	0.041*	
C10	0.94613 (12)	0.32426 (12)	0.3393 (2)	0.0293 (4)	
C11	0.84995 (12)	0.30971 (12)	0.2835 (2)	0.0309 (4)	
H11	0.8095	0.3614	0.2527	0.037*	
C12	0.81509 (13)	0.22161 (13)	0.2739 (2)	0.0352 (4)	
H12	0.75	0.2124	0.237	0.042*	
N2	0.87086 (12)	0.14714 (11)	0.3155 (2)	0.0357 (3)	
N3	0.98294 (12)	0.40973 (11)	0.3540 (2)	0.0368 (4)	
H2	0.8439 (17)	0.0887 (18)	0.322 (3)	0.061 (7)*	
H3A	0.9432 (16)	0.4610 (15)	0.322 (3)	0.045 (6)*	
H3B	1.0408 (17)	0.4127 (15)	0.391 (3)	0.048 (6)*	
O1W	0.8108 (2)	0.52435 (14)	0.8618 (3)	0.1094 (11)	
H1W	0.817 (2)	0.541 (2)	0.965 (5)	0.080 (10)*	
H2W	0.806 (2)	0.562 (2)	0.781 (5)	0.096 (11)*	

### Atomic displacement parameters (Å^2^)

**Table:**

	*U*^11^	*U*^22^	*U*^33^	*U*^12^	*U*^13^	*U*^23^
C1	0.0257 (8)	0.0296 (8)	0.0228 (7)	0.0026 (6)	0.0032 (6)	−0.0001 (6)
C2	0.0310 (8)	0.0261 (8)	0.0245 (7)	0.0017 (6)	0.0048 (6)	0.0017 (6)
C3	0.0268 (8)	0.0306 (8)	0.0281 (8)	−0.0034 (6)	0.0046 (6)	−0.0036 (6)
C4	0.0241 (8)	0.0377 (9)	0.0244 (7)	0.0046 (6)	0.0007 (6)	−0.0018 (6)
C5	0.0319 (9)	0.0339 (9)	0.0312 (8)	0.0036 (7)	0.0030 (7)	0.0082 (7)
C6	0.0265 (9)	0.0340 (9)	0.0325 (8)	−0.0007 (7)	0.0033 (6)	0.0062 (7)
C7	0.0260 (8)	0.0281 (8)	0.0319 (8)	0.0004 (6)	−0.0007 (6)	0.0059 (6)
N1	0.0256 (8)	0.0466 (9)	0.0316 (7)	0.0043 (6)	−0.0001 (6)	−0.0049 (7)
O1	0.0307 (7)	0.0477 (8)	0.0437 (7)	0.0115 (6)	−0.0002 (5)	−0.0040 (6)
O2	0.0367 (7)	0.0580 (8)	0.0302 (6)	−0.0009 (6)	−0.0039 (5)	−0.0001 (6)
O3	0.0254 (7)	0.0589 (9)	0.0530 (8)	−0.0023 (6)	0.0004 (6)	−0.0042 (7)
O4	0.0382 (8)	0.0584 (9)	0.0541 (8)	0.0136 (7)	−0.0054 (6)	0.0154 (7)
Cl1	0.0433 (3)	0.0341 (2)	0.0463 (3)	0.00160 (18)	0.0065 (2)	0.01473 (18)
C8	0.0429 (11)	0.0369 (9)	0.0287 (8)	0.0107 (8)	0.0059 (7)	0.0010 (7)
C9	0.0295 (9)	0.0400 (10)	0.0323 (8)	0.0069 (7)	0.0027 (7)	0.0002 (7)
C10	0.0306 (9)	0.0360 (9)	0.0214 (7)	0.0035 (7)	0.0037 (6)	−0.0002 (6)
C11	0.0282 (9)	0.0381 (9)	0.0262 (8)	0.0056 (7)	0.0033 (6)	0.0018 (7)
C12	0.0316 (9)	0.0472 (10)	0.0266 (8)	−0.0011 (7)	0.0030 (7)	0.0004 (7)
N2	0.0440 (9)	0.0359 (8)	0.0275 (7)	−0.0008 (7)	0.0060 (6)	0.0002 (6)
N3	0.0289 (8)	0.0361 (8)	0.0435 (9)	0.0021 (7)	−0.0018 (7)	−0.0013 (7)
O1W	0.249 (4)	0.0447 (10)	0.0407 (10)	0.0464 (14)	0.0425 (14)	0.0092 (9)

### Geometric parameters (Å, º)

**Table:**

C1—C2	1.395 (2)	C8—N2	1.347 (2)
C1—C6	1.398 (2)	C8—C9	1.353 (3)
C1—C7	1.515 (2)	C8—H8	0.95
C2—C3	1.381 (2)	C9—C10	1.418 (2)
C2—Cl1	1.7369 (16)	C9—H9	0.95
C3—C4	1.381 (2)	C10—N3	1.334 (2)
C3—H3	0.95	C10—C11	1.407 (2)
C4—C5	1.376 (2)	C11—C12	1.358 (2)
C4—N1	1.471 (2)	C11—H11	0.95
C5—C6	1.383 (2)	C12—N2	1.345 (2)
C5—H5	0.95	C12—H12	0.95
C6—H6	0.95	N2—H2	0.93 (3)
C7—O2	1.248 (2)	N3—H3A	0.94 (2)
C7—O1	1.248 (2)	N3—H3B	0.84 (2)
N1—O3	1.219 (2)	O1W—H1W	0.75 (3)
N1—O4	1.230 (2)	O1W—H2W	0.78 (4)
			
C2—C1—C6	118.13 (15)	O4—N1—C4	117.71 (15)
C2—C1—C7	122.05 (14)	N2—C8—C9	121.43 (16)
C6—C1—C7	119.83 (14)	N2—C8—H8	119.3
C3—C2—C1	121.91 (15)	C9—C8—H8	119.3
C3—C2—Cl1	118.28 (12)	C8—C9—C10	119.60 (17)
C1—C2—Cl1	119.80 (12)	C8—C9—H9	120.2
C4—C3—C2	117.63 (15)	C10—C9—H9	120.2
C4—C3—H3	121.2	N3—C10—C11	121.74 (16)
C2—C3—H3	121.2	N3—C10—C9	120.92 (16)
C5—C4—C3	122.84 (15)	C11—C10—C9	117.33 (16)
C5—C4—N1	118.88 (15)	C12—C11—C10	119.83 (16)
C3—C4—N1	118.27 (15)	C12—C11—H11	120.1
C4—C5—C6	118.46 (15)	C10—C11—H11	120.1
C4—C5—H5	120.8	N2—C12—C11	121.31 (17)
C6—C5—H5	120.8	N2—C12—H12	119.3
C5—C6—C1	121.02 (16)	C11—C12—H12	119.3
C5—C6—H6	119.5	C12—N2—C8	120.46 (16)
C1—C6—H6	119.5	C12—N2—H2	118.9 (15)
O2—C7—O1	126.79 (16)	C8—N2—H2	120.3 (15)
O2—C7—C1	116.89 (15)	C10—N3—H3A	118.3 (13)
O1—C7—C1	116.28 (14)	C10—N3—H3B	116.1 (15)
O3—N1—O4	124.01 (15)	H3A—N3—H3B	126 (2)
O3—N1—C4	118.28 (15)	H1W—O1W—H2W	117 (3)
			
C6—C1—C2—C3	0.4 (2)	C2—C1—C7—O1	99.31 (18)
C7—C1—C2—C3	−179.64 (14)	C6—C1—C7—O1	−80.7 (2)
C6—C1—C2—Cl1	179.99 (12)	C5—C4—N1—O3	−179.69 (15)
C7—C1—C2—Cl1	0.0 (2)	C3—C4—N1—O3	−0.8 (2)
C1—C2—C3—C4	−0.6 (2)	C5—C4—N1—O4	−0.2 (2)
Cl1—C2—C3—C4	179.75 (12)	C3—C4—N1—O4	178.68 (15)
C2—C3—C4—C5	0.1 (2)	N2—C8—C9—C10	0.8 (3)
C2—C3—C4—N1	−178.79 (14)	C8—C9—C10—N3	178.61 (16)
C3—C4—C5—C6	0.8 (3)	C8—C9—C10—C11	−1.9 (2)
N1—C4—C5—C6	179.59 (14)	N3—C10—C11—C12	−178.78 (16)
C4—C5—C6—C1	−1.0 (3)	C9—C10—C11—C12	1.8 (2)
C2—C1—C6—C5	0.5 (2)	C10—C11—C12—N2	−0.5 (2)
C7—C1—C6—C5	−179.52 (15)	C11—C12—N2—C8	−0.8 (2)
C2—C1—C7—O2	−82.6 (2)	C9—C8—N2—C12	0.6 (2)
C6—C1—C7—O2	97.40 (18)		

### Hydrogen-bond geometry (Å, º)

**Table:**

*D*—H···*A*	*D*—H	H···*A*	*D*···*A*	*D*—H···*A*
N2—H2···O1*W*^i^	0.93 (3)	1.72 (3)	2.641 (2)	170 (2)
N3—H3*A*···O1	0.94 (2)	1.99 (2)	2.926 (2)	171.1 (19)
N3—H3*B*···O2^ii^	0.84 (2)	2.16 (3)	2.982 (2)	165 (2)
O1*W*—H1*W*···O1^iii^	0.75 (3)	1.96 (3)	2.698 (2)	166 (3)
O1*W*—H2*W*···O2	0.78 (4)	1.99 (4)	2.729 (2)	158 (3)

Symmetry codes: (i) *x*, −*y*+1/2, *z*−1/2; (ii) −*x*+2, −*y*+1, −*z*+1; (iii) *x*, *y*, *z*+1.
